# Effectiveness of Dupilumab in the Treatment of Patients with Severe Uncontrolled CRSwNP: A “Real-Life” Observational Study in the First Year of Treatment

**DOI:** 10.3390/jcm11102684

**Published:** 2022-05-10

**Authors:** Eugenio De Corso, Stefano Settimi, Claudio Montuori, Marco Corbò, Giulio Cesare Passali, Davide Paolo Porru, Simone Lo Verde, Camilla Spanu, Daniele Penazzi, Giuseppe Alberto Di Bella, Eleonora Nucera, Matteo Bonini, Gaetano Paludetti, Jacopo Galli

**Affiliations:** 1 Unit of Otorhinolaryngology—Head and Neck Surgery, A. Gemelli Hospital Foundation IRCCS, 00168 Rome, Italy; eugenio.decorso@policlinicogemelli.it (E.D.C.); giuliocesare.passali@unicatt.it (G.C.P.); gaetano.paludetti@unicatt.it (G.P.); jacopo.galli@iol.it (J.G.); 2 Department of Head and Neck and Sensory Organs, Catholic University of the Sacred Hearth, 00168 Rome, Italy; claudio_montuori@libero.it (C.M.); marco.corbo@icloud.com (M.C.); davide.cp@tiscali.it (D.P.P.); loverde.simo@gmail.com (S.L.V.); camillaspanu@gmail.com (C.S.); daniele.penazzi@gmail.com (D.P.); giuseppealberto.dibella@gmail.com (G.A.D.B.); 3 Unit of Allergology, A. Gemelli Hospital Foundation IRCCS, 00168 Rome, Italy; eleonora.nucera@policlinicogemelli.it; 4 Department of Internal Medicine and Geriatrics, Catholic University of the Sacred Hearth, 00168 Rome, Italy; matteo.bonini@policlinicogemelli.it; 5 Unit of Pulmonology, A. Gemelli Hospital Foundation IRCCS, 00168 Rome, Italy

**Keywords:** chronic rhinosinusitis with nasal polyps, type-2 inflammation, asthma, biologics, dupilumab, real life, eosinophilic otitis media, eosinophils, treatment outcomes

## Abstract

The aim of this study was to evaluate the efficacy of dupilumab in the treatment of severe uncontrolled Chronic Rhinosinusitis with Nasal Polyps (CRSwNP), with or without asthma as add-on therapy with intra-nasal corticosteroids in a real-life setting over the first year of treatment. Our data demonstrated that subcutaneous 300 mg dupilumab administered at home via a pre-filled auto-injector every two weeks, based on indications set by the Italian Medicines Agency, was rapidly effective in reducing the size of polyps, decreasing symptoms of disease, improving quality of life, and recovering olfaction. Significant improvement was observed after only 15 days of treatment, and it progressively increased at 6 and 12 months. Dupilumab was also effective in reducing the local nasal eosinophilic infiltrate, in decreasing the need for surgery and/or oral corticosteroids, and in improving control of associated comorbidities such as chronic eosinophilic otitis media and bronchial asthma. After 12 months of treatment, 96.5% of patients had a moderate/excellent response. From our data, it was evident that there was a group of patients that showed a very early response within one month of therapy, another group with early response within six months from baseline, and a last group that improved later within 12 months. The results of this study support the use of dupilumab as an effective option in the current standard of care for patients affected by severe uncontrolled CRSwNP.

## 1. Introduction

Chronic Rhinosinusitis with Nasal Polyps (CRSwNP) is a difficult to treat pathology and a challenge for the otolaryngologist due to difficulties in therapeutic management of the underlying chronic inflammatory process. In addition, it has a significant negative impact on patients’ quality of life, and affected patients may also present other comorbidities such as asthma, allergic rhinitis, and intolerance to non-steroidal anti-inflammatory drugs (NSAIDs), which contribute to determine the severity of the phenotype [1,2,3].

It is known that there are forms of chronic rhinosinusitis that cannot be controlled with maximal medical therapy and surgical treatment, which were recently defined as “severe uncontrolled CRSwNP” [1,4]. For these patients, therapy with biological drugs (monoclonal antibodies that specifically target the type-2 inflammatory pathway underlying the disease, acting as anti-IL4R, anti-IL5, anti-IL5R, anti-IgE agents) was recently proposed, and some have been approved by US and European regulatory authorities [5]. Among these biologics, dupilumab is a fully human monoclonal antibody that binds the alpha subunit of IL-4 receptors (IL-4Rα type 1 and type 2) to inhibit the signaling of IL-4 and IL-13; it is the first biological drug approved by the FDA for the treatment of CRSwNP (June 26th in 2019) in adults as add-on therapy with intra-nasal corticosteroids (INCS) [5]. Dupilumab also received a favorable opinion on October 26th in 2019 from the European Medicine Agency (EMA) and the Italian Medicines Agency on 9th December 2020. In Italy, dupilumab is prescribed as an add-on therapy with INCS for adult patients affected by severe uncontrolled CRSwNP (defined by a Nasal Polyp Score ≥ 5 or a Sino-Nasal Outcome Test-22 score ≥ 50) who did not achieve control of disease with oral corticosteroids (OCS) and/or surgery [5].

The aim of this study was to evaluate the efficacy of dupilumab in the treatment of severe uncontrolled CRSwNP, with or without asthma, in a real-life setting over the first year of treatment. The primary endpoints were reduction in the Nasal Polyp Score (NPS) and improvement in nasal obstruction, quality of life, and olfactory function (evaluated as early as possible within the first month of treatment and later at 6 and 12 months of treatment). Secondary endpoints were reduction in the local nasal eosinophilic infiltrate, achievement of disease control in terms of need for surgery and/or oral corticosteroids (OCS), and improvement in associated comorbidities such as eosinophilic otitis media (EOM) and bronchial asthma.

## 2. Materials and Methods

### 2.1. Population and Study Design

This is a monocentric observational study in a real-life setting. We included 57 patients (mean age: 51.9 years; range 23–75, F:M = 0.7:1) affected by severe uncontrolled CRSwNP who received, in real-life clinical practice, subcutaneous 300 mg dupilumab administered every 2 weeks with an auto-injector as add-on therapy to INCS. Patients were followed between February 2021 and March 2022 at the A. Gemelli Hospital Foundation-IRCCS, Catholic University of Sacred Heart, Rhinology Unit, Rome, Italy.

Dupilumab was prescribed according to the therapeutic plan set by the Italian Medicines Agency: age of at least 18 years; confirmed diagnosis of diffuse CRSwNP by endoscopy and CT performed at least 6 months before therapy; severe disease stage defined by NPS ≥ 5 or Sino-Nasal Outcome Tests-22 (SNOT-22) ≥ 50; inadequate symptom control with INCS; failure or intolerance of previous medical treatments (at least 2 cycles of systemic corticosteroid in the last year) and/or failure of previous surgical treatment after endoscopic sinus surgery (ESS) with postoperative complications or no clinical benefit. In real-life, we considered the following as exclusion criteria for treatment: pregnancy; immunosuppressive therapy; radio-chemotherapy for cancer in the 12 months before the start of therapy; concomitant long-term corticosteroid therapy for chronic autoimmune disorders.

The study was approved by the local ethics committee (Number of protocol: ID 4429). Informed consent about privacy and utilization of clinical data was obtained from all patients at the time of original data collection. Clinical data were anonymously analyzed.

### 2.2. Methodology and Efficacy Outcomes

In clinical routine practice, based on our institutional protocol, patients were evaluated at baseline (V0) before starting biological therapy and during treatment: more specifically, after 15 days (V1), 1 month (V2), 3 months (V3), 6 months (V4), 9 months (V5), and 12 months (V6) from the first administration.

At baseline and follow–up visits, patients underwent endoscopic evaluation, quality of life assessment, evaluation of nasal obstruction and olfaction, nasal cytology, and symptoms of asthma.

#### 2.2.1. Endoscopic Evaluation

Dimension of polyps was evaluated with the Nasal Polyps Score (NPS): Each side of the nasal cavity was separately evaluated and scored in a range from 0 to 4 (0 = no polyps, 1 = small polyps in the middle meatus not reaching below the inferior border of the middle turbinate, 2 = polyps reaching below the lower border of the middle turbinate, 3 = large polyps reaching the lower border of the inferior turbinate or polyps medial to the middle turbinate, and 4 = large polyps causing complete obstruction of the inferior nasal cavity). The sum of scores for both nasal cavities was recorded as the NPS [6].

#### 2.2.2. Quality of Life Assessment

*SNOT-22*. We used the validated Italian version of SNOT-22 with a possible total score range of 0–110. A SNOT-22 score < 20 was suggestive of mild symptoms. During follow-up, the minimal clinically important difference in SNOT-22 scores was assumed for an 8.9-point increase, as reported in previous studies [7].

*EQ-5D-5L.* This descriptive system comprises five dimensions: mobility, self-care, usual activities, pain/discomfort, and anxiety/depression. Each dimension has 5 levels: no problems, slight problems, moderate problems, severe problems, and extreme problems. The patient is asked to indicate his/her health state by ticking the box next to the most appropriate statement in each of the five dimensions. We took into consideration the EQ-VAS, which records the respondent’s overall current health in a vertical visual analogue scale from 0 to 100 points, where the endpoints are labeled “The best health you can imagine” (100 points) and “The worst health you can imagine” (0 points). The EQ-VAS provides a quantitative measure of the patients’ perception of their overall health [8].

*VAS symptoms*. Intensity of symptoms was measured on a horizontal 10 cm line. A mean score for each symptom analyzed was obtained using the average value of the scores assigned for the same symptom [9].

*Total Nasal Symptom Score (TNSS).* The possible score was 0–15; it is the sum of 5 individual participant-assessed symptom scores for rhinorrhea, nasal congestion, nasal itching, sneezing, and difficulty sleeping, each evaluated using a scale of 0 = None, 1 = Mild, 2 = Moderate, or 3 = Severe [10].

#### 2.2.3. Evaluation of Nasal Obstruction

*Nasal Congestion Score (NCS).* Patients evaluated their symptoms of congestion/obstruction from the previous day using the NC scale 0: no symptoms; 1: mild symptoms (symptoms clearly present, but minimal awareness and easily tolerated); 2: moderate symptoms (definite awareness of symptoms that are bothersome but tolerable); 3: severe symptoms (symptoms that are hard to tolerate, cause interference with activities of daily living) [11].

*Peak Nasal Inspiratory Flow (PNIF).* PNIF was measured to assess the degree of nasal obstruction. For the evaluation, we used the PNIF-meter, a simple-to-use instrument with proven diagnostic validity, which measures the PNIF through the nasal cavity, providing an objective value of the degree of nasal obstruction. Values between 80 L/min and 200 L/min are considered normal, with an average physiological value of approximately 140 L/min [9].

#### 2.2.4. Olfactory Evaluation

*VAS olfaction.* Intensity of symptom (hyposmia) was measured on a horizontal 10 cm line [9].

*Sniffin’ sticks-16 Identification test (SSIT-16)*. This test is performed by presenting 16 odors at suprathreshold intensity to the patient who must identify each by choosing from the four options provided. Depending on the number of correctly identified substances, a result between 0 (no substance identified) and 16 (all substances identified) is obtained. This allowed us to classify patients as anosmic (score between 0 and 5), hyposmic (score between 6 and 10), or normosmic (score between 11 and 16) [12,13,14].

*Chemosensory Complaint Score-CCS.* The questionnaire gives two sub-scores relating to olfactory (Smell Complaint Score, SCS) and gustatory (Taste Complaint Score, TaCS) dysfunction. The total score of CCS (TCS) is the sum of SCS and TaCS; it may vary from 0 to 16: the lower the score obtained, the lower the impact that the dysfunction has on the patient’s life [15].

#### 2.2.5. Asthma Symptoms

*ACT score*. A patient self-administered tool for identifying those with poorly controlled asthma. ACT assesses the frequency of shortness of breath and general asthma symptoms, use of rescue medications, effect of asthma on daily functioning, and overall self-assessment of asthma control. It consists of a 5-point scale (for symptoms and activities: 1 = all the time to 5 = not at all; for asthma control rating: 1 = not controlled at all to 5 = completely controlled). The scores range from 5 (poor control of asthma) to 25 (complete control of asthma), with higher scores reflecting greater asthma control. An ACT score > 19 indicates well-controlled asthma [16].

#### 2.2.6. Local Inflammation Assessment

*Nasal cytology and eosinophilic infiltrate reduction*. Nasal leukocyte counts were performed on nasal scraped tissue, obtained from the inferior turbinate bilaterally. Scraping was performed with a rhinoprobe (Farmark s.n.c, Milan, Italy) as in our previous experience [17,18,19]. The sample was gently spread on glass slides and immediately fixed in 95% ethyl alcohol and stained with May-Grunwald-Giemsa. The percentage of eosinophils was assessed by microscopic cytological examination. The slides were examined under oil immersion by light microscopy first at a magnification of 400× and then at a magnification of 1000×. Eosinophil counts were expressed as a percentage of cells of granulocytic or mononuclear cells, excluding nasal epithelial ones, at a high power field, as the mean of at least 10 fields observed. Nasal tissue eosinophil infiltration was measured as “Eosinophil count per high power field (Ec-hpf)” and reported as the mean of at least 3 richest high-powered fields observed at nasal cytology [1,2,20].

#### 2.2.7. Evaluation of Disease Control by EPOS Criteria

Based on EPOS criteria [1], we divided patients according to treatment clinical response as follows: (a) NPS reduction (at least 1 point); (b) SNOT-22 reduction (at least 8.9 points); (c) OCS need reduction; (d) Sniffin’ Sticks-16 identification test improvement (at least 4 points); (e) Reduced impact of comorbidities. Based on the above criteria, the patients were divided into 4 groups: “no responder” (0 criteria met); “Poor responder” (1–2 criteria met); “Moderate responder” (3–4 criteria met); and “Excellent responder” (5 criteria met) [1].

At 12 months of treatment, patients were considered eligible to remain on treatment with dupilumab if the following criteria were satisfied, according to the EUFOREA indication [4]: NPS < 4; SNOT < 30; VAS < 5; NCS < 2.

Table 1 summarizes the baseline characteristics of the cohort.

### 2.3. Statistical Analysis

The analysis was performed using SPSS for Windows (IBM Corp, Chicago, IL, USA). Normality of continuous variables was verified with the Shapiro–Wilk test (normal for *p* > 0.05). The *t*-test for paired samples was used for normally distributed data. The Mann–Whitney U-test was used for non-normally distributed data. All results are reported as mean ± standard deviation (SD). Statistical significance was assumed for *p*-values < 0.05. All comparisons were made between data obtained at different follow-up times (example, 6 months after the beginning of therapy) and baseline.

## 3. Results

### 3.1. Efficacy of Dupilumab on NPS Reduction and Restoring Nasal Obstruction

Dupilumab was shown to be effective in reducing NPS and restoring nasal obstruction (measured by the Nasal Congestion Score and PNIF). The mean NPS score decreased significantly from 5.7 ± 1.56 at baseline to 3.85 ± 1.72 at 15 days of treatment (*p <* 0.05), to 2.53 ± 1.72 at 6 months (*p <* 0.05), and to 1.81 ± 1.75 at 12 months (*p <* 0.05). The mean NCS score decreased significantly from 2.38 ± 0.85 at baseline to 1.32 ± 0.77 at 15 days of treatment (*p* < 0.05), to 0.64 ± 0.55 at 6 months (*p* < 0.05), and to 0.61 ± 0.50 at 12 months (*p* < 0.05).

**Figure 1:**
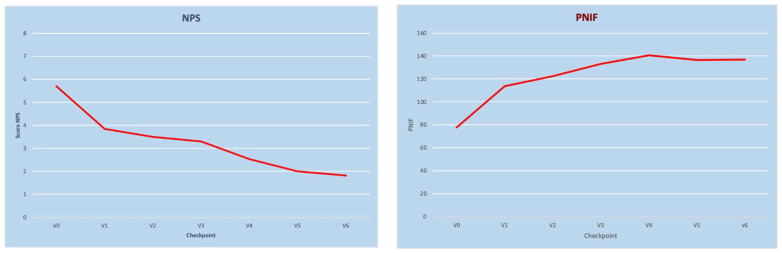
NPS (**left panel**) and PNIF (**right panel**) mean value variations over time. (NPS: nasal polyp score; PNIF: peak nasal inspiratory flow; V0: visit at baseline; V1: 15 days of treatment; V2: 1 month visit; V3: 3-month visit; V4: 6-month visit; V6: 12-month visit).

Accordingly, a significant improvement was also found with objective measures of nasal obstruction with PNIF: the mean PNIF improved from 77.8 ± 45.4 L/min at baseline to 113.75 ± 46.71 at 15 days of treatment (*p* < 0.05), to 140.7 ± 43.17 L/min at 6 months (*p* < 0.05), and to 136.9 ± 39.46 at 12 months (*p* < 0.05). The temporal modification of mean NPS and PNIF scores over the first year of treatment is shown in Figure 1.

### 3.2. Efficacy of Dupilumab on Quality of Life and Olfactory Function

In our series, we observed significant improvement in quality of life measured with several indicators. We observed an average reduction in SNOT-22 from 59.56 ± 19.56 at baseline to 34.02 ± 20.66 after the first injection measured at 15 days of treatment (*p* < 0.05). The mean SNOT-22 further decreased to 19.5 ± 15.98 at 6 months of treatment and to 10.8 ± 9.29 at 12 months (*p* < 0.05). Furthermore, patients reported an improvement in perception of good health and well-being measured with EQ-VAS: the mean composite score improved from 66.44 ± 19.13 at baseline to 72.35 ± 15.27 at day 15 of treatment (*p* < 0.05), to 81.57± 12.96 at 6 months (*p* < 0.05), and to 81.76 ± 12.0 at 12 months of treatment (*p* < 0.05). Regarding the TNSS, the mean score decreased from 13.27 ± 4.16 at baseline to 5.71 ± 3.78 at 15 days (*p* < 0.05), to 4.66 ± 4.49 at 6 months (*p* < 0.05), and to 2.23 ± 1.59 at 12 months of treatment (*p <* 0.05).

A general improvement in olfaction measured with the Sniffin’ Sticks-16 Identification test, VAS olfaction, and the CCS score was observed as early as 15 days of treatment and further improved until 12 months of treatment. The SSIT-16 mean score improved from 3.83 ± 3.2 at baseline to 7.57 ± 3.9 at 15 days of treatment. This positive trend, starting from the first drug administration, was confirmed at 4 weeks with an increase in olfactory performance to 8.29 ± 4.89 at the mean SSIT-16. The latter further improved to 10.85 ± 1.77 at 6 months (*p <* 0.05) and to 11.12 ± 1.67 at 12 months (*p <* 0.05).

Table 2 shows the distribution of patients based on results with the SSIT-16 during treatment.

In addition, the CCS olfaction mean score decreased from 6.3 ± 3.8 at baseline to 3.5 ± 3.0 at 6 months (*p* < 0.05) and to 0.7 ± 1.0 at 12 months (*p* < 0.05).

**Figure 2:**
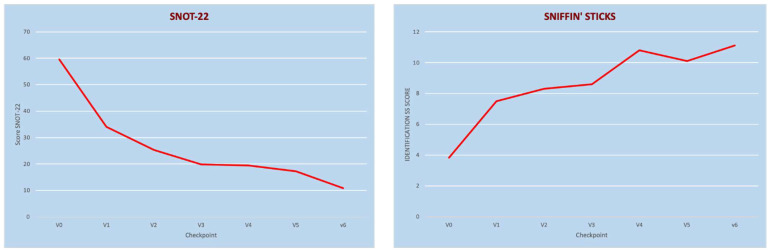
SNOT-22 (**left panel**) and Sniffin’Sticks-16 Identification Test (**right panel**) over time. V0: visit at baseline; V1: 15 days of treatment; V2: 1 month; V3: 3 months of treatment; V4: 6 months of treatment; V6: 12 months of treatment).

Mean VAS olfaction values decreased from 8.49 ± 1.96 at baseline to 5.79 ± 3.52 at 15 days (*p* < 0.05), to 2.56 ± 2.42 at 6 months (*p* < 0.05), and to 2.42 ± 2.27 at 12 months (*p* < 0.05). Mean values of the SNOT-22 and Sniffin’ Sticks-16 Identification Test over the first year are shown in Figure 2.

### 3.3. Efficacy of Dupilumab on Local Eosinophilic Inflammation

Dupilumab was shown to be effective in reducing local eosinophilic inflammation in most patients. In particular, 73.6% (42/57) of patients had local eosinophilic inflammation with a cell count greater than >10 hpf. After 3 months of treatment, 13/57 (22.8%) still had a positive nasal cytology for eosinophilic inflammation (*p* < 0.05); at 6 months, 9/57 patients (15.8%) had local inflammation at nasal cytology (*p* < 0.05). At 12 months, none of the patients had local inflammation at nasal cytology (*p* < 0.05).

Table 3 shows the main outcomes at baseline and during treatment.

### 3.4. Efficacy of Dupilumab on Disease Control in Terms of Need for OCS and Surgery, and Associated Comorbidities

At baseline, patients had been administered a mean number of 3.05 short cycles of OCS in the last year. Simultaneously with the beginning of biological therapy, they stopped OCS, being only administered with dupilumab as add-on therapy to INCS. In addition, they never had the need for OCS during treatment with dupilumab.

The same trend was observed for surgery. At baseline, 48/57 (84.2%) patients had undergone at least 1 previous surgery for CRSwNP. During treatment with dupilumab, surgery was not needed in any case. On the other hand, 9/57 (15.7%) patients did not undergo surgery before dupilumab treatment because they were not fit for surgery due to an anesthesiologic contraindication.

Regarding associated comorbidities and the impact of dupilumab on lung function, we observed improvement in the ACT score during treatment. At baseline, patients had an average ACT score of 17.44 ± 5.53. We observed significant progressive improvement in the mean score at 6 and 12 months, increasing to 22.51 ± 2.06 (*p* < 0.05) and 23.7 ± 2.16 (*p* < 0.05), respectively.

Eosinophilic otitis media (EOM) was observed in 3/57 of our patients. We noticed a reduction in EOM associated symptoms (evaluated with the Otitis Severity Score proposed by Iino et al. [21] and the Italian validated version of COMOT-15 [22]) and an improvement in pure tone audiometry-evaluated pure tone average (PTA), as recently published [23].

### 3.5. Evaluation of Disease Control by EPOS Criteria

**Figure 3:**
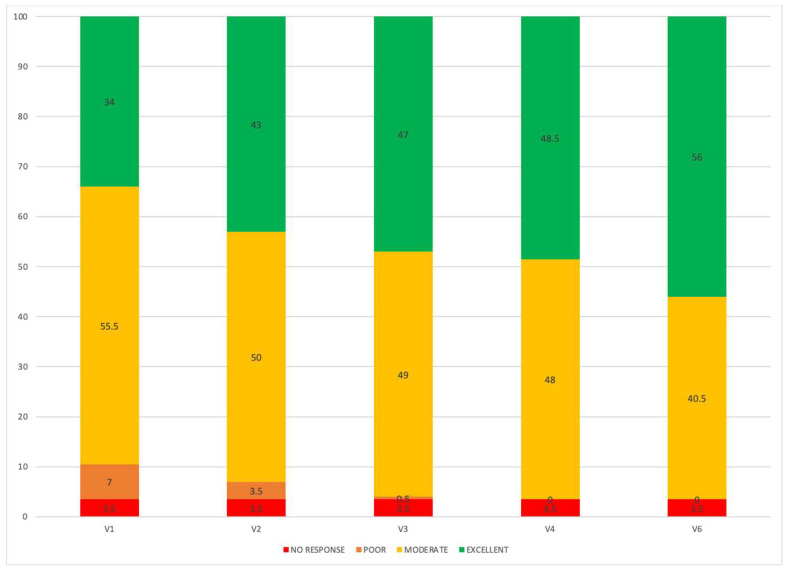
Percentage of response according to EPOS 2020 criteria over the first year of treatment. V0: visit at baseline; V1: 15 days of treatment; V2: 1 months; V3: 3 months; V4: 6 months; V6: 12 months).

We evaluated clinical response, based on EPOS criteria, at each visit during treatment. Two patients had no clinical response during the first year of treatment, meeting none of the criteria proposed by EPOS guidelines (3.5%), and interrupted the treatment at 12 months. In Figure 3, we report the percentage of responses over time.

**Figure 4:**
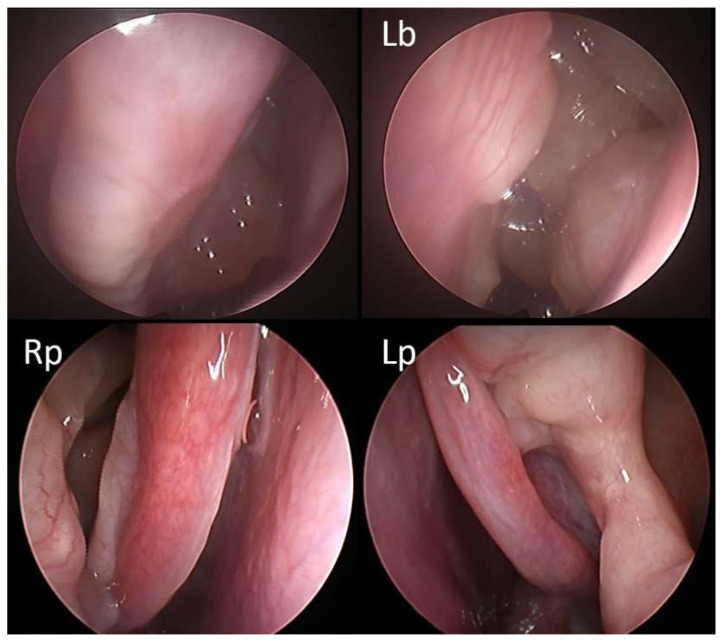
31-year-old male patient with medical history of multiple long-lasting cycles of OCS in the last 2 years (>60 cumulative days/year) and subsequent insulin-resistance and hyperglycemia; two previous surgeries with poor adherence to local corticosteroids. At baseline, the NPS was 5/8 (Rb: right side at baseline; Lb: left side at baseline). Fifteen days after the first administration of dupilumab, polyps were no longer visible (Rp: right side post therapy; Lp: left side post therapy).

**Figure 5:**
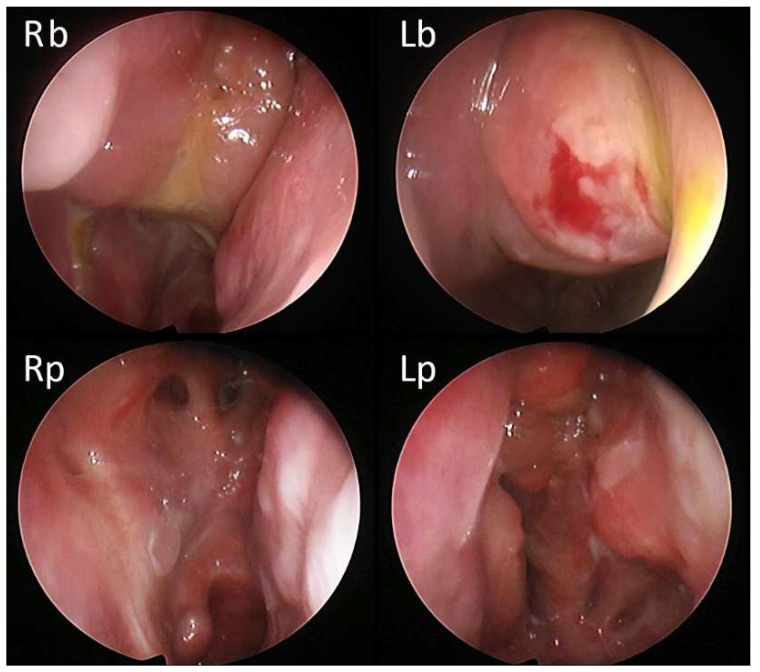
61-year-old female patient with history of 6 previous surgeries, the last complicated with unilateral ophthalmoplegia and vision loss. At baseline, the NPS was 5/8 (Rb: right side at baseline; Lb: left side at baseline). After one month of therapy with dupilumab, the NPS decreased to 1/8 (Rp: right side post therapy; Lp: left side post therapy).

**Figure 6:**
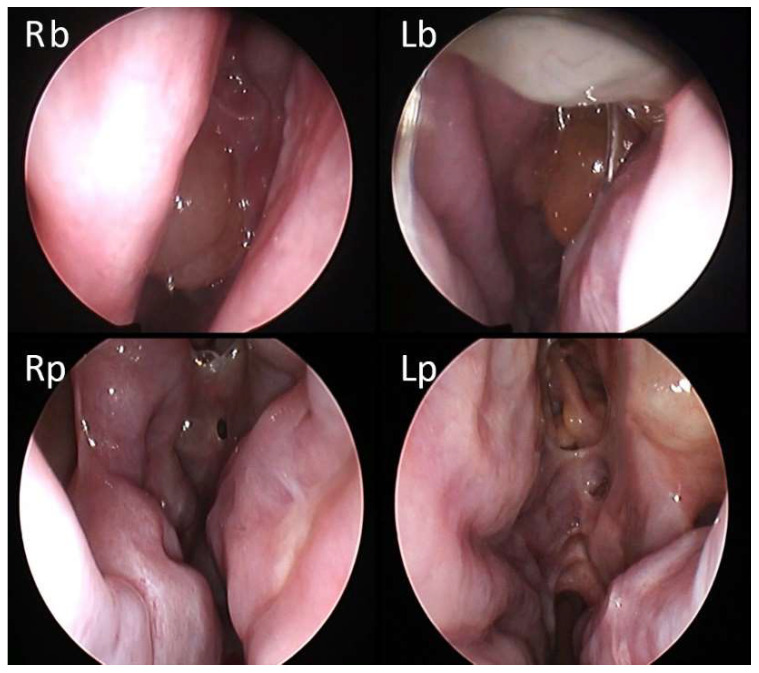
46-year-old male patient with medical history of 5 previous surgeries and persistent mixed neutrophilic eosinophilic infiltration at nasal cytology. Affected by severe OSAS and obesity, the patient was at increased anesthesiologic risk for a new surgery. At baseline, the NPS was 5/8 (Rb: right side at baseline; Lb: left side at baseline). After one month of therapy with dupilumab, polyps showed complete regression (Rp: right side post therapy; Lp: left side post therapy).

At 12 months of treatment, according to EUFOREA criteria 2021 [4], 96.5% of patients were eligible to continue dupilumab therapy. Of note, 12/57 patients (21%) already satisfied the criteria at 1 month of therapy and were considered as super early responders (Figure 4, Figure 5 and Figure 6). In addition, 21/57 patients (37%) satisfied the same criteria within 6 months, and 22/57 patients (38.5%) satisfied the criteria between the 6th and 12th months of treatment.

### 3.6. Safety

Regarding adverse effects, dupilumab was well tolerated by all patients in the study. No severe adverse reactions were reported: one patient reported the onset of migraine after the second drug injection, which resolved within 24 h without medication; 3 subjects reported minor symptoms such as conjunctivitis, which occurred within the first month of treatment, with spontaneous resolution and without the need for medical treatment. A transient increase in the blood eosinophilic count was observed in 18/57 (31.5%) patients after 4 weeks of treatment with stabilization and/or resolution and no adverse effects during 12 months of therapy.

## 4. Discussion

CRSwNP is an inflammatory disorder that includes a variety of phenotypes and affects patients’ quality of life, with a burden of disease that has significant healthcare-related costs [4]. For years, the treatment of CRSwNP was based on medical therapy, using INCS and nasal irrigations with saline solution with or without antihistamines/antileukotrienes; in case of non-response to local therapeutic regimens, short cycles of OCS, with or without antibiotics, can be used to control obstructive nasal symptoms and to reduce the size of polyps volumetrically [3,24,25].

In cases of insufficient control with medical therapy, endoscopic surgical treatment is considered as a valid option to improve nasal obstruction, restore normal ventilation, and improve access for future subsequent local treatments. However, a consistent group of patients do not experience relief with OCS and/or surgery, showing persistence or recurrence of disease [5,26,27]. These patients were recently identified as affected by “severe uncontrolled CRSwNP” [1,3].

Starting from the assumption that the pathophysiology of CRSwNP is driven by eosinophilic inflammation, with related T-helper cell 2 cytokines and IgE formation [17,18,19], biological therapy with monoclonal antibodies used in diseases such as asthma or atopic dermatitis (which are known to have an underlying type 2 inflammatory pathway) can be used for type 2 CRSwNP as well, targeting specific immunologic mediators that are at the basis of the underlying inflammatory process: anti-IL-4/IL-13 signaling (dupilumab), anti-IL-5 pathways (mepolizumab, benralizumab), and anti-IgE antibodies (omalizumab) [5].

The efficacy of dupilumab was demonstrated in a series of clinical trials. In a phase II, randomized, double-blind, placebo-controlled study, Bachert and colleagues [28] evaluated the efficacy of dupilumab in CRSwNP refractory to INCS, with the dupilumab treated group showing significant reduction in polyp size starting from week 4 of treatment [28]. Subsequently, two phase 3 studies, SINUS-24 and SINUS-52 [29], demonstrated the efficacy and safety of subcutaneous dupilumab 300 mg administered every 2 weeks versus placebo in severe uncontrolled CRSwNP. Patients obtained significant improvements in all primary and secondary endpoints (nasal congestion/obstruction severity, NPS, sinus opacification, and loss of smell) at week 24 and 52 [29]. More specifically, for NPS and NCS, significant improvement was observed at week 2, with continued improvement up to the end of treatment in both studies for all endpoints. For loss of smell, 62% of patients treated with dupilumab changed their smell status from anosmic to non-anosmic. Lastly, dupilumab treatment resulted in a significant reduction in OCS use and need for revision surgery compared to placebo [29]. Supporting dupilumab’s mechanism of action, analyses of biomarkers in patients treated with dupilumab in SINUS-52 showed a consistent decrease in concentrations of serum total IgE, periostin, TARC, and plasma eotaxin-3 at weeks 24 and 52 and in levels of ECP, total IgE, eotaxin-3, and IL-5 in nasal secretions at week 24. Furthermore, in SINUS-24, the suspension of dupilumab at week 24 led to loss of efficacy on all endpoints up to 12 months [5].

In our study, from February 2021, we began prescribing dupilumab to patients affected by severe uncontrolled CRSwNP in routine clinical practice. Most of our patients had undergone at least one previous surgery (84.2%), whereas, in the remaining, biological therapy was indicated because these patients were not fit for surgery. However, outcomes between these two groups could not be compared due to the large difference in numbers of patients.

Our data demonstrated that subcutaneous 300 mg dupilumab administered at home via a pre-filled auto-injector every two weeks (based on Italian Medicines Agency indications) is rapidly effective in severe uncontrolled CRSwNP. We observed improvement during therapy with dupilumab in all primary endpoints after only 2 weeks of treatment. More specifically, dupilumab was effective at 2 weeks of treatment in significantly reducing the NPS score, SNOT-22 score, and NCS score. Furthermore, it was effective in significantly improving the PNIF and EQ-VAS. Dupilumab was also effective in a rapid recovery of olfaction as documented by significative improvement in the Sniffin’ Sticks Identification test, VAS olfaction, and CCS olfaction at 2 weeks of treatment. An improvement was also observed in all secondary endpoints: inducing remission of sino-nasal eosinophilic inflammation (as documented by nasal cytology) and reducing OCS and need for surgery during the first year of treatment. In addition, dupilumab was effective in improving comorbidities, restoring lung function (as shown by improved asthma symptoms in the 38 asthmatic patients), and improving ear symptoms and hearing function in the three patients who had comorbid EOM. This trend was observed during 12 months of treatment.

Few authors have evaluated the efficacy of dupilumab in patients with CRSwNP in a real-life setting. Van der Lans et al. [30] reported their preliminary findings of a real-life, prospective observational cohort (*n* = 131) of adults with CRSwNP administered subcutaneous dupilumab 300 mg every 2 weeks. They reported that add-on dupilumab therapy was highly effective in difficult-to-treat type-2 inflammation driven CRSwNP, applying EPOS2020 criteria for biological treatment. Similarly to van der Lans et al., we observed that the therapeutic effects of dupilumab were comparable or slightly favorable in “real-life” compared to LNPS-trials (mainly depending on NPS). In our series, a mean NPS of 2.5 was observed at 6 months, and, in van der Lans’ cohort, a mean six-month NPS of 1.56 was observed, whereas it was 3.75 and 4.46 in LNPS-52 and LNPS-24, respectively. Comparing these results, it should be considered that our cohort is based on Italian Medicines Agency indications, which differ slightly from van der Lans et al.’s [30] experience, who mainly adopted the EPOS2020 indication. Furthermore, in van der Lans’ experience, an interdose interval prolongation of 2 weeks was applied, and more specifically in patients with moderate to excellent response at 6 months of therapy, according to the “stepwise interdose interval prolongation” successfully explored in the Sinus 52 trial. At our institution, the administration plan never changed over the first year of treatment since the adherence rate was very high.

The strength of our study lies in the real-life context in which we standardized indication criteria, treatment regimen, and follow-up schedule. Therapeutic outcomes were monitored throughout the first year of treatment. In this way, it was possible to verify that most patients had significant improvement immediately after the first and second administrations and that the improvement was progressive up to 12 months of therapy. It should be noted that we had the opportunity to observe the rapidity of action of dupilumab in real-life also because we assisted the patients during the first month of treatment, mainly to train them to auto-inject the drug.

Regarding the timing of response, based on EPOS criteria [1] we documented that at 6 months of treatment, 49.12% of patients had an excellent response, even if at 1 month of therapy 43% of patients had a “very early” excellent response. Overall, 96.5% of patients had a moderate/excellent response at 12 months.

All these patients met 2021 EUFOREA criteria [4] to continue treatment with dupilumab. Adopting more restrictive criteria, we tried to apply 2021 EUFOREA criteria [4] not only at 12 months of treatment, but even before. Interestingly, we observed that 21% of patients had satisfied the criteria at 1 month of therapy and 37% of patients at 6 months. From our data, it is evident that there is a group of patients that shows a super early response, another group an early response within six months from baseline, and a group that satisfied the criteria later (38.5%). There is no homogeneous and standardized way to classify the response according to the time of treatment, and this is the major limitation to compare our results with the other series in the literature. Nevertheless, we believe that future multicentric studies on a larger number of patients in a real-life setting could confirm our data, providing the basis to build a clearer definition of early or late responders and even of “super responders”.

Some limitations of this study should be considered: this study was conducted in a tertiary referral center, by reporting results of our first cohort of patients, which could possibly include patients with the most severe and difficult-to-treat CRSwNP. Future inclusion of non-academic patient cohorts will clarify if on a large national scale some differences may be seen; we believe that interesting information could be obtained by comparing different subtypes of severe CRSwNP patients (i.e., patients with NSAID intolerance and asthma compared to the remaining patients in the study) on larger national and even international series. This observational cohort study confirms that dupilumab add-on therapy is highly effective in the management of difficult-to-treat type-2 inflammation driven CRSwNP, validating the criteria set by the Italian Medicines Agency for biological treatment with dupilumab.

## 5. Conclusions

In conclusion, we observed significant and rapid improvement in all efficacy outcomes in patients with severe uncontrolled CRSwNP treated with dupilumab as an add-on therapy to INCS. Dupilumab was effective in reducing the size of polyps and disease-related symptoms, including the improvement in olfaction. Treatment with dupilumab also reduced the need for OCS and surgery and improved comorbidities such as asthma and EOM. The results of this study therefore support the use of dupilumab as an effective new option in the standard of care for patients with severe uncontrolled CRSwNP.

## Figures and Tables

**Table 1:** Baseline characteristics.

	Number or Mean Score ± Standard Deviation	(%)
Number of patients	57	
Age in year (mean age)	51.98 ± 13.12	
Male	34/57	61.4%
Female	23/57	40.3%
**Evidence of type 2 inflammation**		
Asthma	38/57	67%
NSAIDs intolerance	17/57	30%
Peripheral blood hypereosinophilia (>250)	41/57	71.9%
Local Eosinophilia at nasal cytology	42/57	73.7%
NSAID intolerance and asthma	13/57	23%
FeNO (20)	30/57	52.6%
IgE (>100)	20/57	35%
**Staging**		
Mean CT Lund Mackay score	17.8 ± 4.1	
Mean SNOT-22	59.56 ± 19.56	
Mean NPS	5.7 ± 1.56	
Mean PNIF	77.8 ± 45.4	
Mean Sniffin’ Sticks Identification test score	3.83 ± 3.2	
**Control of disease**		
Mean of number of short OCS cycles in the last year	3.05	
Previous surgery	48/57 (84.2%)	
ESS = 0	9/57 (15.8%)	
ESS = 1	16/48 (33.3%)	
ESS > 1	32/48 (66.7%)	

Abbreviations. NSAIDs: non-steroidal anti-inflammatory drugs; FeNO: fractional exhaled nitric oxide; IgE: Immunoglobulin-E; CT: computerized tomography; SNOT-22: sinonasal outcome test-22; NPS: nasal polyp score; PNIF: peak nasal inspiratory flow; OCS: oral corticosteroids; ESS: endoscopic sinus surgery.

**Table 2:** Patient distribution based on results with the SSIT-16 over time.

	Baseline	1 Month	3 Months	6 Months	12 Months
Anosmic	70.2% (40/57)	19.3% (11/57)	17.5% (10/57)	3.5% (2/57)	3.5% (2/57)
Hyposmic	29.8% (17/57)	38.6% (22/57)	38.6% (22/57)	43.8% (25/57)	29.8% (17/57)
Normosmic	0	42.1% (24/57)	43.8% (25/57)	52.6% (30/57)	66.7% (38/57)

**Table 3:** Clinical outcomes during treatment.

	Baseline	3 Months	6 Months	9 Months	12 Months
Mean SNOT-22	59.56 ± 19.56	19.9 ± 13.8	19.5 ± 15.9	17.2 ± 13.1	10.8 ± 9.2
Mean NPS	5.7 ± 1.56	3.3 ± 1.7	2.5 ± 1.5	2 ± 1.7	1.81 ± 1.7
Mean PNIF	77.8 ± 45.4	133 ± 41.3	140.7 ± 43.2	136.6 ± 33.9	136.9 ± 39.6
Mean NCS	2.38 ± 0.85	0.56 ± 0.73	0.64 ± 0.60	0.52 ± 0.49	0.61 ± 0.63
Mean Sniffin’ Sticks-16 IT	3.83 ± 3.2	8.6 ± 4.7	10.8 ± 1.7	10.1 ± 3.1	11.12 ± 1.67
Mean TNSS	13.27 ± 4.16	4.98 ± 3. 08	4.66 ± 4.49	2.44 ± 2.08	2.23 ± 1.59
Mean EQ VAS	66.44 ± 19.13	80.6 ± 13.3	81.57 ± 12.96	79.08 ± 15.5	81.76 ± 13.0
Mean eosinophilic blood count	0.64	1.35	0.73	0.65	0.54
Patients with eosinophil inflammation at nasal cytology	42/57 (73.7%)	13/57 (22.8%)	9/57 (15.8%)	5/57 (8.7%)	0/57
VAS olfaction	8.5 ± 1.9	3.0 ± 3.2	2.5 ± 2.2	2.4 ± 2.2	2.9 ± 2.3
VAS obstruction	7.7 ± 2.1	1.5 ± 1.4	1.4 ± 1.5	1.6 ± 1.9	1.5 ± 1.4
VAS rhinorrhea	6.7 ± 2.6	1.5 ± 1.4	1.4 ± 1.5	1.5 ± 1.4	0.8 ± 0.7
CCS olfaction	6.3 ± 3.8	3.2 ± 3.0	3.5 ± 3.2	2.5 ± 3.1	0.7 ± 1.0

Abbreviations. SNOT-22: sinonasal outcome test-22; NPS: nasal polyp score; PNIF: peak nasal inspiratory flow; NCS: nasal congestion score; VAS: visual analogue scale; CCS: chemosensory complaint score; TNSS: total nasal symptom score.

## Data Availability

The data presented in this study are available on reasonable request from the corresponding author.

## References

[1] Fokkens W.J., Lund V.J., Hopkins C., Hellings P.W., Kern R., Reitsma S., Toppila-Salmi S., Bernal-Sprekelsen M., Mullol J., Alobid I. (2020). European position paper on rhinosinusitis and nasal polyps 2020. Rhinology.

[2] De Corso E., Lucidi D., Battista M., Romanello M., De Vita C., Baroni S., Autilio C., Galli J., Paludetti G. (2017). Prognostic value of nasal cytology and clinical factors in nasal polyps development in patients at risk: Can the beginning predict the end?. Int. Forum Allergy Rhinol..

[3] Hellings P., Akdis C., Bachert C., Bousquet J., Pugin B., Adriaensen G., Advani R., Agache I., Anjo C., Anmolsingh R. (2017). EUFOREA Rhinology Research Forum 2016: Report of the brainstorming sessions on needs and priorities in rhinitis and rhinosinusitis. Rhinol. J..

[4] Bachert C., Han J.K., Wagenmann M., Hosemann W., Lee S.E., Backer V., Mullol J., Gevaert P., Klimek L., Prokopakis E. (2021). EUFOREA expert board meeting on uncontrolled severe chronic rhinosinusitis with nasal polyps (CRSwNP) and biologics: Definitions and management. J. Allergy Clin. Immunol..

[5] De Corso E., Bellocchi G., De Benedetto M., Lombardo N., Macchi A., Malvezzi L., Motta G., Pagella F., Vicini C., Passali D. (2022). Biologics for severe uncontrolled chronic rhinosinusitis with nasal polyps: A change management approach. Consensus of the Joint Committee of Italian Society of Otorhinolaryngology on biologics in rhinology. Acta Otorhinolaryngol. Ital..

[6] Gevaert P., Calus L., Van Zele T., Blomme K., De Ruyck N., Bauters W., Hellings P., Brusselle G., De Bacquer D., van Cauwenberge P. (2013). Omalizumab is effective in allergic and nonallergic patients with nasal polyps and asthma. J. Allergy Clin. Immunol..

[7] Gallo S., Russo F., Mozzanica F., Preti A., Bandi F., Costantino C., Gera R., Ottaviani F., Castelnuovo P. (2020). Prognostic value of the Sinonasal Outcome Test 22 (SNOT-22) in chronic rhinosinusitis. Acta Otorhinolaryngol. Ital..

[8] Kind P., Hardman G., Leese B. (2005). Measuring health status: Information for primary care decision making. Health Policy.

[9] Scadding G., Hellings P., Alobid I., Bachert C., Fokkens W., van Wijk R.G., Gevaert P., Guilemany J., Kalogjera L., Lund V. (2011). Diagnostic tools in Rhinology EAACI position paper. Clin. Transl. Allergy.

[10] Stuyt J.A.G., Luk L., Keschner D., Garg R. (2021). Evaluation of In-Office Cryoablation of Posterior Nasal Nerves for the Treatment of Rhinitis. Allergy Rhinol..

[11] Linder A. (1988). Symptom scores as measures of the severity of rhinitis. Clin. Exp. Allergy.

[12] Passali G.C., Passali D., Cingi C., Ciprandi G. (2022). Smell impairment in patients with chronic rhinosinusitis: A real-life study. Eur. Arch. Oto-Rhino-Laryngol..

[13] Hummel T., Whitcroft K., Andrews P., Altundag A., Cinghi C., Costanzo R., Damm M., Frasnelli J., Gudziol H., Gupta N. (2017). Position paper on olfactory dysfunction. Rhinol. J..

[14] Hummel T., Kobal G., Gudziol H., Mackay-Sim A. (2007). Normative data for the “Sniffin’ Sticks” including tests of odor identification, odor discrimination, and olfactory thresholds: AN upgrade based on a group of more than 3,000 subjects. Eur. Arch. Oto-Rhino-Laryngol..

[15] Heald A.E., Pieper C.F., Schiffman S.S. (1998). Taste and smell complaints in HIV-infected patients. AIDS.

[16] Huvanandana J., Nguyen C.D., Foster J.M., Frey U., Reddel H.K., Thamrin C. (2021). Novel Methods of Measuring Adherence Patterns Reveal Adherence Phenotypes with Distinct Asthma Outcomes. Ann. Am. Thorac. Soc..

[17] De Corso E., Baroni S., Lucidi D., Battista M., Romanello M., Autilio C., Morelli R., Di Nardo W., Passali G.C., Sergi B. (2015). Nasal lavage levels of granulocyte-macrophage colony-stimulating factor and chronic nasal hypereosinophilia. Int. Forum Allergy Rhinol..

[18] De Corso E., Baroni S., Battista M., Romanello M., Penitente R., Di Nardo W., Passali G.C., Sergi B., Fetoni A.R., Bussu F. (2014). Nasal fluid release of eotaxin-3 and eotaxin-2 in persistent sinonasal eosinophilic inflammation. Int. Forum Allergy Rhinol..

[19] De Corso E., Baroni S., Romitelli F., Luca L., Di Nardo W., Passali G.C., Paludetti G. (2011). Nasal lavage CCL24 levels correlate with eosinophils trafficking and symptoms in chronic sino-nasal eosinophilic inflammation. Rhinol. J..

[20] Gelardi M., Iannuzzi L., De Giosa M., Taliente S., De Candia N., Quaranta N., De Corso E., Seccia V., Ciprandi G. (2017). Non-surgical management of chronic rhinosinusitis with nasal polyps based on clinical-cytological grading: A precision medicine-based approach. Acta Otorhinolaryngol. Ital..

[21] Iino Y., Tomioka-Matsutani S., Matsubara A., Nakagawa T., Nonaka M. (2011). Diagnostic criteria of eosinophilic otitis media, a newly recognized middle ear disease. Auris Nasus Larynx.

[22] Cavaliere M., Capriglione P., Cavaliere F., De Corso E., Zanoletti E., Motta G., Iengo M., Cantone E. (2021). Cross-cultural adaptation and Italian validation of chronic otitis media outcome test 15 (COMOT-15). Acta Otorhinolaryngol. Ital..

[23] De Corso E., Montuori C., Settimi S., Mele D.A., Cantiani A., Corbò M., Cantone E., Paludetti G., Galli J. (2022). Efficacy of Biologics on Refractory Eosinophilic Otitis Media Associated with Bronchial Asthma or Severe Uncontrolled CRSwNP. J. Clin. Med..

[24] De Corso E., Settimi S., Tricarico L., Mele D.A., Mastrapasqua R.F., di Cesare T., Salvati A., Trozzi L., De Vita C., Romanello M. (2021). Predictors of Disease Control After Endoscopic Sinus Surgery Plus Long-Term Local Corticosteroids in CRSwNP. Am. J. Rhinol. Allergy.

[25] De Corso E., Anzivino R., Galli J., Baroni S., Di Nardo W., De Vita C., Salvati A., Autilio C., Settimi S., Mele D. (2019). Antileukotrienes improve naso-ocular symptoms and biomarkers in patients with NARES and asthma. Laryngoscope.

[26] Bachert C., Bhattacharyya N., Desrosiers M., Khan A.H. (2021). Burden of Disease in Chronic Rhinosinusitis with Nasal Polyps. J. Asthma Allergy.

[27] Vlaminck S., Acke F., Prokopakis E., Speleman K., Kawauchi H., van Cutsem J.-C., Hellings P.W., Jorissen M., Seys S., Bachert C. (2021). Surgery in Nasal Polyp Patients: Outcome After a Minimum Observation of 10 Years. Am. J. Rhinol. Allergy.

[28] Bachert C., Mannent L., Naclerio R.M., Mullol J., Ferguson B.J., Gevaert P., Hellings P., Jiao L., Wang L., Evans R.R. (2016). Effect of Subcutaneous Dupilumab on Nasal Polyp Burden in Patients With Chronic Sinusitis and Nasal Polyposis. JAMA.

[29] Bachert C., Han J.K., Desrosiers M., Hellings P.W., Amin N., Lee S.E., Mullol J., Greos L.S., Bosso J.V., Laidlaw T.M. (2019). Efficacy and safety of dupilumab in patients with severe chronic rhinosinusitis with nasal polyps (LIBERTY NP SINUS-24 and LIBERTY NP SINUS-52): Results from two multicentre, randomised, double-blind, placebo-controlled, parallel-group phase 3 trials. Lancet.

[30] Van der Lans R.J.L., Fokkens W.J., Adriaensen G.F.J.P.M., Hoven D.R., Drubbel J.J., Reitsma S. (2021). Real-life observational cohort verifies high efficacy of dupilumab for chronic rhinosinusitis with nasal polyps. Allergy.

